# Congenital Syphilis, Réunion Island, 2010

**DOI:** 10.3201/eid1711.101925

**Published:** 2011-11

**Authors:** Juliana Ramiandrisoa, Lydéric Aubert, Emilie Boidin Lespine, Jean-Luc Alessandri, Pierre-Yves Robillard, Marc Bertsch, Anne Gallay, Véronique Goulet, Eric D’Ortenzio

**Affiliations:** Regional Office of French Institute for Public Health Surveillance in the Indian Ocean, Saint-Denis, Réunion Island, France (J. Ramiandrisoa, L. Aubert, E.B. Lespine, E. D’Ortenzio); Regional Hospital, Saint-Denis (J.-L. Alessandri, P.-Y. Robillard); Centre Hospitalier Gabriel Martin, Saint-Paul, Réunion Island (M. Bertsch); French Institute for Public Health Surveillance, Saint-Maurice, France (A. Gallay, V. Goulet)

**Keywords:** syphilis, Treponema pallidum, congenital, Indian Ocean, Réunion Island, mass screening, pregnancy, bacteria, letter

**To the Editor**: Syphilis, caused by the bacterium *Treponema pallidum*, is primarily a sexually transmitted infection, but *T. pallidum* can also be transmitted by infected pregnant women to their children. Every year, at least 500,000 children are born with congenital syphilis (CS); maternal syphilis causes another half million stillbirths and abortions, usually in countries with limited resources (*1*). However, CS has been recently found in industrialized countries such as the United States, where the CS rate increased by 23% during 2005–2008, after a 38% increase in the syphilis rate among US women and girls during an earlier period (2004–2007) (*2*).

Réunion Island, a French overseas territory with 810,000 inhabitants, has a health care system similar to that in continental France. Neither syphilis infection, CS, nor other trepanomatosis (yaws) is notifiable. Since 2006, an increase in early syphilis was documented, first in men who have sex with men infected with HIV and second in the general population.

In 2009, we conducted a retrospective study by using data from 2004–2009 to document the situation of CS on the island. Data from all public (n = 4) and private (n = 2) hospitals on the island with neonatology and obstetrical departments were investigated. Birth deliveries at home were not included. Inclusion criteria were positive specific (*T. pallidum* hemagglutination assay) and nonspecific (Venereal Disease Research Laboratory [VDRL]) test results for *Treponema* spp. among children <2 years of age during 2004–2009. Additionally, hospitalized children coded as having congenital syphilis (International Classification of Diseases [ICD] 10 codes A50.0 to A50.9) in the French national hospital database were included. After reviewing medical files of mothers and their children, cases were classified as confirmed or probable CS according to the case definition of the Centers for Disease Control and Prevention (*2*).

Eighteen children had positive syphilis serologic results by *T. pallidum* hemagglutination assay and VDRL tests, according to the selection criteria. Among these 18 test results, 7 were classified as probable CS (late treatment for mother or symptoms linked to CS), 3 in 2008 and 4 in 2009 (Table). The male:female sex ratio was 0.75. Five case-patients were preterm newborns; 3 of the most premature babies had signs linked to CS, such as hepatosplenomegaly, cutaneous mucosal signs, neurologic signs, radiographic signs of CS in long bones, edema, and biologic anomalies. All were screened for *T. pallidum*–specific IgM by using fluorescent treponemal antibody absorption or IgM capture ELISA from immediately after birth to 15 days old. Two case-patients had positive results; 1 was symptomatic. Six of the 7 children who had probable CS received appropriate penicillin G treatment, except for 1 asymptomatic baby for whom long-term medical supervision was recommended by the pediatrician. Survival rates at 3 months of age reached 100%.

**Table:** Clinical and biological characteristics of mothers and children with congenital syphilis, Réunion Island, 2010*

Year of diagnosis	Mother						Child	
	Age, y	Time of syphilis screening	Duration of treatment, d	Presence andsatage of disease	Positive serologic titer test results		Gestation, wk	Clinical signs
2008	16	>13 wk gestation	5 d BD	Probable secondary syphilis at first trimester	TPHA, VDRL, FTA-ABS		34	None
2008	25	Unknown	2 d AD	NA	TPHA, VDRL, FTA-ABS		34	None
2008	16	AD	14 d AD	Primary syphilis at third trimester	TPHA, VDRL, FTA-ABS		38	None
2009	26	>13 wk gestation	17 d BD	NA	TPHA, VDRL, FTA-ABS		31	Present
2009	18	>13 wk gestation	2 d BD	NA	TPHA, VDRL		32	Present
2009	22	>13 wk gestation	1 d BD	NA	TPHA, VDRL		32	Present
2009	37	After delivery	1 d AD	NA	TPHA, VDRL		38	None

*BD, before delivery; TPHA, *Treponema pallidum* hemagglutination assay; VDRL, Venereal Disease Research Laboratory; FTA-ABS, fluorescent treponemal antibody absorption; AD, after delivery; NA, not applicable.

Median age of mothers at delivery was 22 years. All mothers were natives of Réunion Island except 1 who was born in Madagascar and received no antenatal follow-up. Medical history indicated previous genital herpes for 3 women. Social difficulties or alcohol consumption were reported for 3 women. The mean age of gestation at which the first syphilis screening was conducted was 23 weeks (5–33 weeks). Two mothers were symptomatic. Syphilis was diagnosed after delivery for 3 mothers; seroconversion occurred during the pregnancy. Except for missing data on 1 mother, all mothers were HIV negative.

In Réunion Island, in our retrospective review, we found 7 CS cases during 2008–2009 but none during 2004–2007. The incidence rate of probable CS cases was estimated to be 28 cases per 100,000 live births during 2009. However, results may have been underestimated because not all parturients with a positive syphilis test result and fetal deaths were investigated. Meanwhile, a fetal death at 30 weeks was reported during the investigation but not included in the selection criteria. The Centers for Disease Control and Prevention definition of CS based on maternal status can also lead to an overestimation. Late screening of syphilis in mothers, lack of antenatal follow-up, higher VDRL titer, or unknown stage of the disease at time of diagnosis have already been described in other studies (*3*–*5*).

Our report highlights an alarming situation in Réunion Island. Reemergence of CS after the increase of early syphilis in women of childbearing age must be considered as a public health alert, especially in countries where health care is supposed to be efficient. CS is easy to prevent with adequate screening of the mother and good follow-up of seropositive parturients.

The results of our study permitted reinforcement of the syphilis mass screening and awareness campaign regarding this sexually transmitted infection in the general population and medical corps. Although it is unrealistic to expect complete eradication of primary and secondary syphilis in communities, a minimal increase of CS rates should trigger reinforcement of these prevention policies.
